# Reducing the effective dose of doxycycline using chitosan silver nanocomposite as a carriers on gram positive and gram-negative bacteria

**DOI:** 10.1038/s41598-024-78326-1

**Published:** 2024-11-13

**Authors:** Elham M. Mostafa, Y. Badr, M. M. Hashem, K. Abo-EL-Sooud, Amna H. Faid

**Affiliations:** 1 Department of Laser Sciences and Interactions, National Institute of Laser Enhanced Science (NILES), Cairo University, Giza, Egypt; 2 Department of Pharmacology, Faculty of Veterinary Medicine, Cairo University, Giza, Egypt

**Keywords:** Silver nanoparticle, Green Synthesis, Doxycycline, Nanocomposite, Antibacterial effect, Biochemistry, Drug discovery, Microbiology

## Abstract

Doxycycline (Doxy) is a tetracycline antibiotic with a potent antibacterial activity against a broad range of bacteria. Using nanotechnology is one feasible way to increase the antibiotics’ ability to penetrate the body and increase their antibacterial effectiveness. In this work, we report the formation of a stable green synthesized silver nanoparticles (AgNPs) by chitosan with Doxy nanocomposite for the first time. The obtained nanoparticles were characterized by transmission electron microscopy (TEM), zeta-potential, UV-Visible spectroscopy and four transform infrared spectroscopy (FTIRs). The antibacterial effect of doxy, AgNPs and doxy/AgNPs were determined on Gram-positive *Staphylococcus aureus*, *Streptococcus mutans* and Gram-negative *Escherichia coli*, *Klebsiella pneumonia*. This combined therapeutic agent restored the susceptibility of doxy and showed an antibacterial activity against tested bacteria. AgNPs has absorption peak at 445 nm, mixing of Doxy with AgNPs causes all doxy absorptions to red shift and a broadening in surface plasmon resonance (SPR) for AgNPs and show a slight increase in particle size of AgNPs from 12 ± 2 nm to 14 ± 2 nm with high stability as zeta potential was 29 mv and 48.5mv for AgNPs and Doxy/AgNPs respectively. The antibacterial effect of Doxy/AgNPs nanocomposite was found to be twice effect of free doxy, suggesting a synergistic interaction between the two components. In conclusion, synergy of doxy with AgNPs is quite promising for antibiotic resistant strains. These results highlight the ability of AgNPs to boost the efficacy of the doxycycline.

## Introduction

The development of antimicrobial resistance means antibiotics are becoming ineffective in an increasing number of infections. Previous publication in The Lancet in 2022 found that 4.95 million people died from cases related to drug-resistant bacterial infection^1^.

Antibiotics play a crucial role in preventing and treating infectious diseases in both humans and animals. However, the overuse of antibiotics can lead to the development of resistant bacteria, which can then spread from animals to humans. Additionally, the presence of antibiotic residues in livestock raises concerns about potential outbreaks of infectious diseases^2–4^. Therefore, it is essential to explore alternative strategies that can reduce antibiotic usage while maintaining their effectiveness to prevent future pandemics. Nanotechnology has emerged as a promising solution to combat antibiotic resistance, with nano catalysts and drug delivery systems offering significant advantages in overcoming drug resistance^5–9^. Antibiotics exhibit significant biological activities against harmful microorganisms and are widely regarded as the most effective drugs for improving human health^3,4,8–13^. In addition to their primary application, antimicrobials are also utilized in the prevention and treatment of various infections in animals, thereby promoting growth in animal farming. Doxycycline (Doxy), a member of the tetracycline family, is considered an extensive-spectrum antibiotic effective against both Gram-positive and Gram-negative bacteria. It is extensively employed in the treatment of malaria in humans and avian respiratory tract diseases^14–18^. However, the excessive use of Doxy not only contributes to bacterial resistance but also poses risks to human health, including liver damage, allergic reactions, gastrointestinal disturbances, and tooth discoloration. Moreover, the widespread use of this antibiotic has raised concerns as it can enter sewage water and subsequently contaminate the ground and drinking water globally, as they are rarely completely removed. To mitigate the potential hazards associated with excessive antibiotic use, it is crucial to implement stringent monitoring and detection measures, even for trace amounts of antibiotics^12,13,19^. There are certain situations where combination antimicrobial therapy can offer benefits, such as treating mixed bacterial infections that are not responsive to a single antimicrobial treatment, achieving synergistic antimicrobial effects against highly resistant strains like *Pseudomonas aeruginosa*, or minimizing the risk of bacterial resistance. Typically, combination therapy is most effective for mixed infections or when a quick decision needs to be made in a critical situation. Throughout history, silver has been recognized as one of the most potent natural antiseptic elements^20^. The chemical properties of Ag + ions are responsible for the antibacterial properties of nano silver, allowing them to effectively kill microorganisms through various mechanisms^17,21^. As a result, microorganisms rarely develop resistance to nano silver. At the nanometer size, silver nanoparticles (AgNPs) can easily enter bacterial cell walls and generate reactive oxygen species, which disrupt the replication of DNA^22–24^. Consequently, they disable the protective mechanisms such as efflux pumps and biofilm barriers that contribute to microbial resistance against conventional antibiotics^25,26^. Ultimately, these actions can lead to the death of the microorganisms. Metal nanoparticles represent one of the most promising categories of nanomaterials due to their altered physical and chemical properties, which facilitate a diverse array of applications in pharmaceuticals, biosensing, biomedical engineering, and nanomedicine^27,28^. These nanoparticles can serve as carriers for antimicrobials, participate in drug delivery systems, or, in the case of silver nanoparticles, function as antimicrobials on their own. Additionally, AgNPs offer other advantages such as inhibiting the growth of multiple pathogens, including viruses, bacteria, and fungi^28–30^. A potential alternative compound that can be used in silver nanoparticles production is a weak reducing agent. Several scientists have explored the use of weak reducing agent such as trinatrium citrate, chitosan, and glucose^31–33^. Chitosan, among the numerous biopolymers found in nature, has garnered significant interest due to its natural antimicrobial characteristics. Furthermore, chitosan is recognized as a non-toxic, biodegradable polymer that exhibits excellent biocompatibility, minimal immune response, as well as mucoadhesive and absorption-enhancing capabilities. Owing to these advantageous traits, chitosan is extensively utilized in drug delivery systems (DDS), which offer enhanced biodistribution, greater specificity, improved sensitivity, and reduced pharmacological toxicity^34,35^. However, despite these appealing attributes, chitosan presents certain limitations, including its inadequate mechanical strength and rapid degradation rate. To address these challenges, it is often cross-linked, combined with other natural polymers, or incorporated into composites. The various properties of chitosan play a crucial role in determining its effectiveness in the development of innovative, multifunctional DDS^36^. Chitosan is particularly interesting as it not only acts as a reductor but also as a stabilizing agent. Previous studies have successfully utilized chitosan in the silver nanoparticles creation^15,16^. When chitosan solution is used, the silver metal nanoparticles become dispersed, resulting in a colloidal system known as silver-chitosan colloidal nanocomposite^4,11,15,16^. In previous studies, chitosan nanoparticles were used for Doxycycline encapsulation. Moreover, AgNPs were prepared via different reducing agent such as chemical reducing agent, plant extract with subsequent loading with Doxycycline. Additionally, certain antibiotics, including cefotaxime and cephalexin, can function as reducing agents, facilitating the formation of stabilized metal nanoparticles from metal ions^37^. In this study, a novel nanocarrier system was developed by loading Doxycycline on AgNPs reduced by chitosan. The purpose of this combination was to broaden the antibacterial spectrum, minimize the risk of resistance strains, and achieve a rapid and potent bactericidal effect.

## Materials and methods

### Biosynthesis of AgNPs by chitosan reduction

In the present experiment, the synthesis of AgNPs was done according to the method described in previous literature^4,15,16,38^, with a slight modification to obtain a narrower size distribution. Briefly, 1 g of chitosan medium molecular weight (99.9%, Sigma-Aldrich) was dissolved in a 1% acetic acid (v/v) solution. 8 ml silver nitrate solution (8.89 mg/ml) (99.9%, Sigma-Aldrich) mixed with 20 ml of chitosan solution (6.92 mg/ ml) and stirred until homogenous for 30 min. Next, the above mixture was transferred to a beaker and left at 95 °C for 12 h. Within a few hours after the reaction, the solution transformed from colorless to light yellow and lastly to yellowish brown, demonstrating the creation of AgNPs .

### Preparation of doxycycline loaded AgNPs (Doxy/AgNPs Nanocomposite)

Conjugation of Doxy/AgNPs Nanocomposite was attained by mixing 2 ml of the ninth of the two fold dilution 1.9 µg/ ml doxycycline to 2 ml of the AgNPs the sixth of two fold dilution (1.2 µg/ml) with continuous stirring for 15 min forming 0.95 µg/ml Doxy/AgNPs Nanocomposite^39^.

### Antimicrobial test

Using the agar well diffusion technique, the produced nanoparticles’ antibacterial effectiveness was evaluated^40^. Using nutritional agar media, the synthesized nanoparticles were tested in vitro against Gram-positive bacteria *(Streptococcus mutans)*, Gram-negative bacteria *(Klebsiella pneumonia)*, and Gram-positive bacteria *(Staphylococcus aureus)*. The usual medications for Gram-positive and Gram-negative bacteria were ampicillin and gentamicin, respectively. The solvent used as the negative control was DMSO. The compounds were evaluated against the bacterial strains at a concentration of 15 mg/ml.

### Method of testing

Sterilized media was transferred into sterilized Petri dishes (20–25 ml per dish) and permitted to harden at room temperature. A microbial suspension was prepared in sterilized saline equivalent to McFarland 0.5 standard solution (1.5 × 105 CFU mL^−1^) with turbidity adjusted to OD = 0.13 via a spectrophotometer at 625 nm. Within 15 min of altering the suspension turbidity, a sterile cotton swab was dipped into the suspension and spread on the dried agar surface, followed by a 15-minute drying period with the lid in place. Wells of 6 mm diameter were created in the solidified media by means of a sterile borer. 100 µL of the prepared nanoparticles solution was added to each well then incubated at 37 °C for 24 h to evaluate the antibacterial activity. Experiment was conducted in triplicate, and the zones of inhibition were measured in millimeters.

### Statistical analysis

Variances among samples of the same bacterial type were examined via one-way analysis of variance (ANOVA), after that the Duncan multiple comparisons test with SPSS package version “22” for Windows. The experiment was repeated three times independently, and each concentration was repeated three times. Results are presented as mean ± S.E., with *p* < 0.05 considered statistically significant, *p* < 0.01 highly significant, and *p* < 0.001 very highly significant.

### Minimum inhibitory concentrations (MICs) and minimum bactericidal concentrations (MBCs) determination of Doxy/AgNPs nanocomposite

The MIC and MBC of Doxy/AgNPs was assessed using sterile 96-well plates, in accordance with the Clinical and Laboratory Standards Institute^41^. The bacteria were grown on Muller Hinton Broth (MHB), then diluted to 10^6^ CFUmL^−1^in the same medium. Several dilutions of Doxycycline (1000, 500, 250, 125, 62.5, 31.25, 15.6, 7.8, 3.9, 1.9 µg/ml), native AgNPs (78.4, 39.2, 19.6, 9.8, 4.9, 2.45, 1.2, 0.6, 0.3, 0.15 μm/ml) and Doxy/AgNPs ( 500, 250, 125, 62.5, 31.25, 15.6, 7.8, 3.9, 1.9, 0.95 µg/ml). An aliquot of 100 µL of each solution were poured in the wells of the 96-well plates, to which 100 µL of diluted bacterial suspension were added. Each peptide concentration test was repeated in three consecutive wells. The plates were then incubated for 18 h at 37 °C. The bacterial growth was quantified using an Uv-Visible power wave microplate spectrophotometer (BioTech, Vermont, USA) at 620 nm. A column in the plate was used as positive control, where the wells containing 100 µL MHB were inoculated with 100 µL bacterial suspension without antimicrobial agents. Another column was used for negative control, where 100 µL of MHB was added alone. MBCs were determined by taking 10 µL from clear wells and cloudy positive control wells, which were seeded on sterile agar medium and incubated for 24 h at 37 °C. The concentration that causes 0.1% live cells was considered as the MBC value^42^.

### Characterization of Doxy/AgNPs nanocomposite

#### UV-Vis spectroscopy measurements

That was recorded via Cary 5000 (UV-Vis spectrophotometer, Varian) in a wavelength ranging from 200 to 800 nm.

#### Transmission Electron microscope (TEM)

A high-resolution transmission electron microscope (HR-TEM, Tecnai G20, FEI, Netherlands) was used for imaging. A drop of diluted sample solution was placed on a copper grid coated with amorphous carbon. Bright-field imaging mode was done with an Eagle CCD camera using a lanthanum hexaboride (LaB6) electron source gun and an electron acceleration voltage of 200 kV.

#### Dynamic light scattering

The measurements were performed via a Zeta-Sizer Nano-ZS (Malvern Instruments Ltd, UK) equipped with a 633 nm laser. This analysis provides the size distribution and polydispersity index (PDI) of particles within the sample. The hydrodynamic particle diameter can be obtained by the Stokes-Einstein equation.

#### Fourier Transform Infrared Spectroscopy (FTIRs)

FTIRs were performed between 500 and 4000 cm^−1^ via an FTIR spectrometer (4100 Jasco, Japan), by means of a lyophilizer, AgNPs, and AgNPs@Cs were freeze-dried.

## Results and discussion

### Characterization of AgNPs and Doxy/AgNPs

#### Ultra-violet visible spectroscopy

**Fig. 1:**
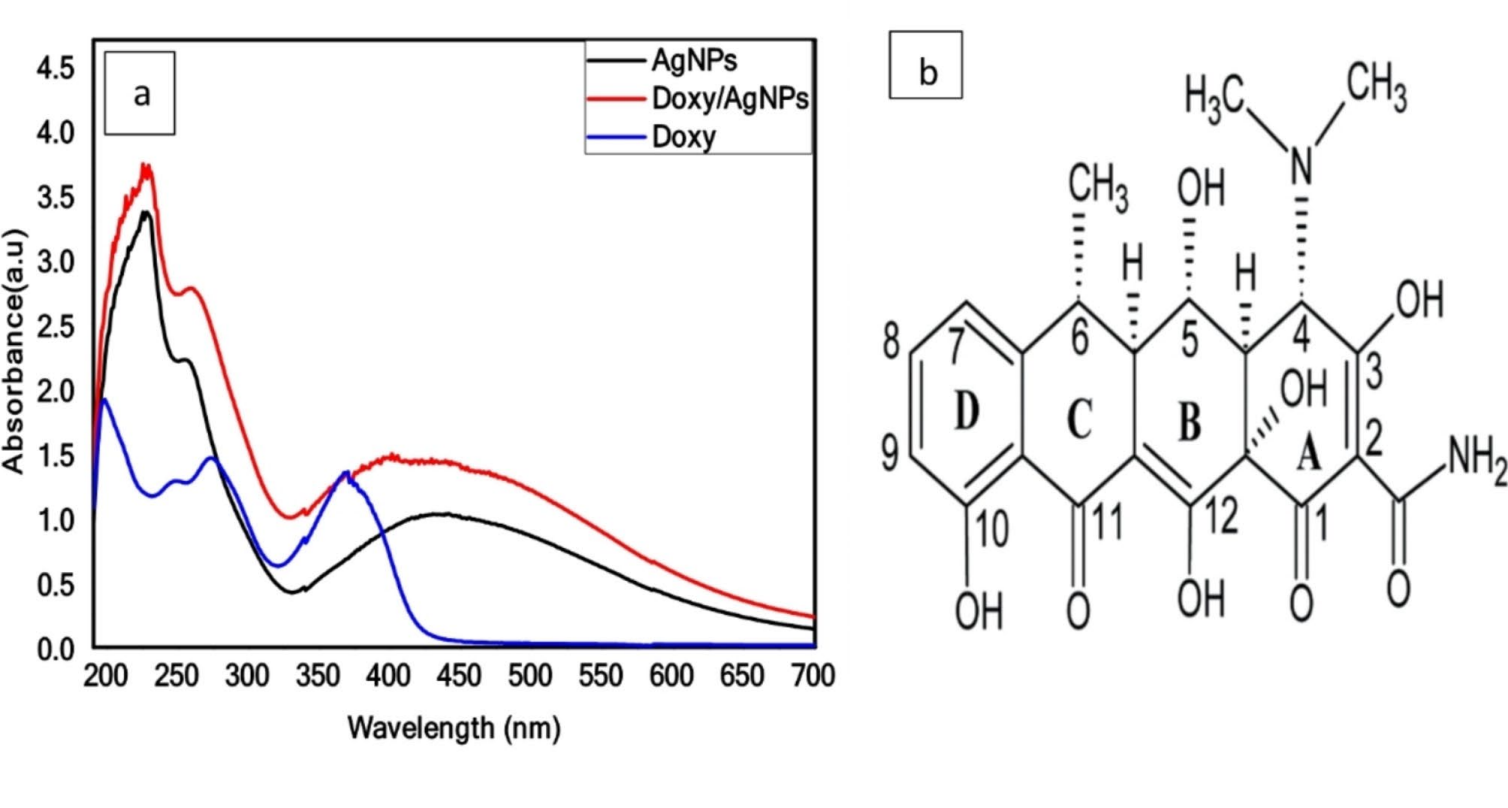
(**a**) UV-Visible absorbance spectra of AgNPs, Doxy and Doxy/AgNPs and (**b**)Chemical structure of doxycycline.

Chitosan, derived through the deacetylation process of chitin, serves as a bio polysaccharide found in the structural polymer of arthropod exoskeletons. Its appeal in nanoparticle production has grown due to its biodegradable nature, non-toxic properties, and cost-effectiveness, surpassing traditional reducing chemicals like formamide, sodium borohydride, and hydrazine. The individual Doxy exhibits two pronounced UV-Vis absorption peaks at 352 nm and 270 nm. This spectroscopical fingerprint was associated to π-π* electronic transitions. The formation of the Doxy/AgNPs nanocomposite was verified by analyzing the UV-Visible absorption spectra, as depicted in Fig. (1**a**,** b).**The UV-Visible spectrum of pure Doxy (blue curve) displays a peak at 370 nm, attributed to the π-electron system in keto–enol system found in ring A. On the other hand, AgNPs exhibit a peak at 445 nm, conforming the characteristic Surface Plasmon Resonance (SPR) of chitosan-stabilized spherical AgNPs. The combination of Doxy with AgNPs (red curve) leads to a red shift in all Doxy absorptions and a broadening of the SPR band for AgNPs, along with a slight red shift to 450 nm indicating an interaction between the system and the AgNPs^6^. The obtained results are consistent with recent reports^37^.

#### Transmission electron microscope (TEM)

**Fig. 2:**
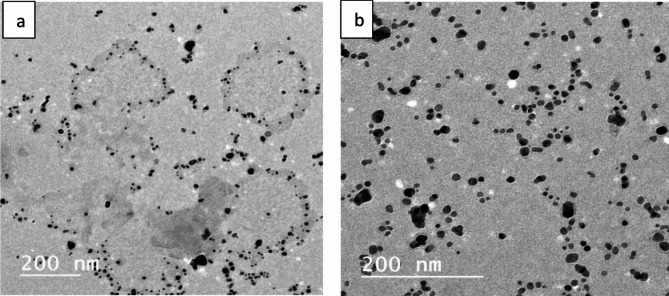
TEM images of (**a**) AgNPs and (**b**) Doxy/AgNPs.

TEM images of AgNPs and Doxy/AgNPs are shown in Fig. (2**a**,** b)** revealed that the prepared AgNPs and Doxy/AgNPs nanocomposite are spherical non-aggregated particles with a metallic core and homogenous size distribution. Upon the conjugation of Doxy with AgNPs a slight increase in particle size of AgNPs from 12 ± 2 nm to 14 ± 2 nm without impact on shape which demonstrate the formation of nanocomposite.

#### Zeta potential analysis

**Fig. 3:**
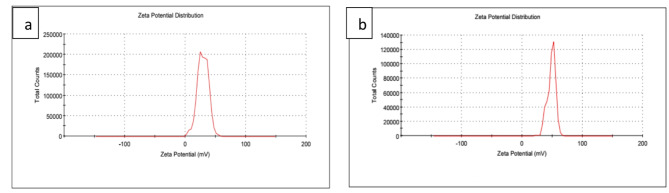
zeta potential of (**a**) AgNPs and (**b**) Doxy/AgNPs.

The zeta potential is a measure of the electric charge between colloidal particles, representing the total charge accumulated on the surface of the particles. Colloidal particles which have low zeta potential value tend to attract each other and yield aggregates. On the other hand, a high zeta potential leads to the formation of a highly stable colloid^43^. Zeta potential of AgNPs and Doxy/AgNPs were examined, Fig. (3**a and b)** shows zeta potential were 29 mv and 48.5mv for AgNPs and Doxy/AgNPs respectively indicating the high stability of the prepared AgNPs and Doxy/AgNPs nanocomposite.

#### Fourier Transform Infrared Spectroscopy (FTIRs)

**Fig. 4:**
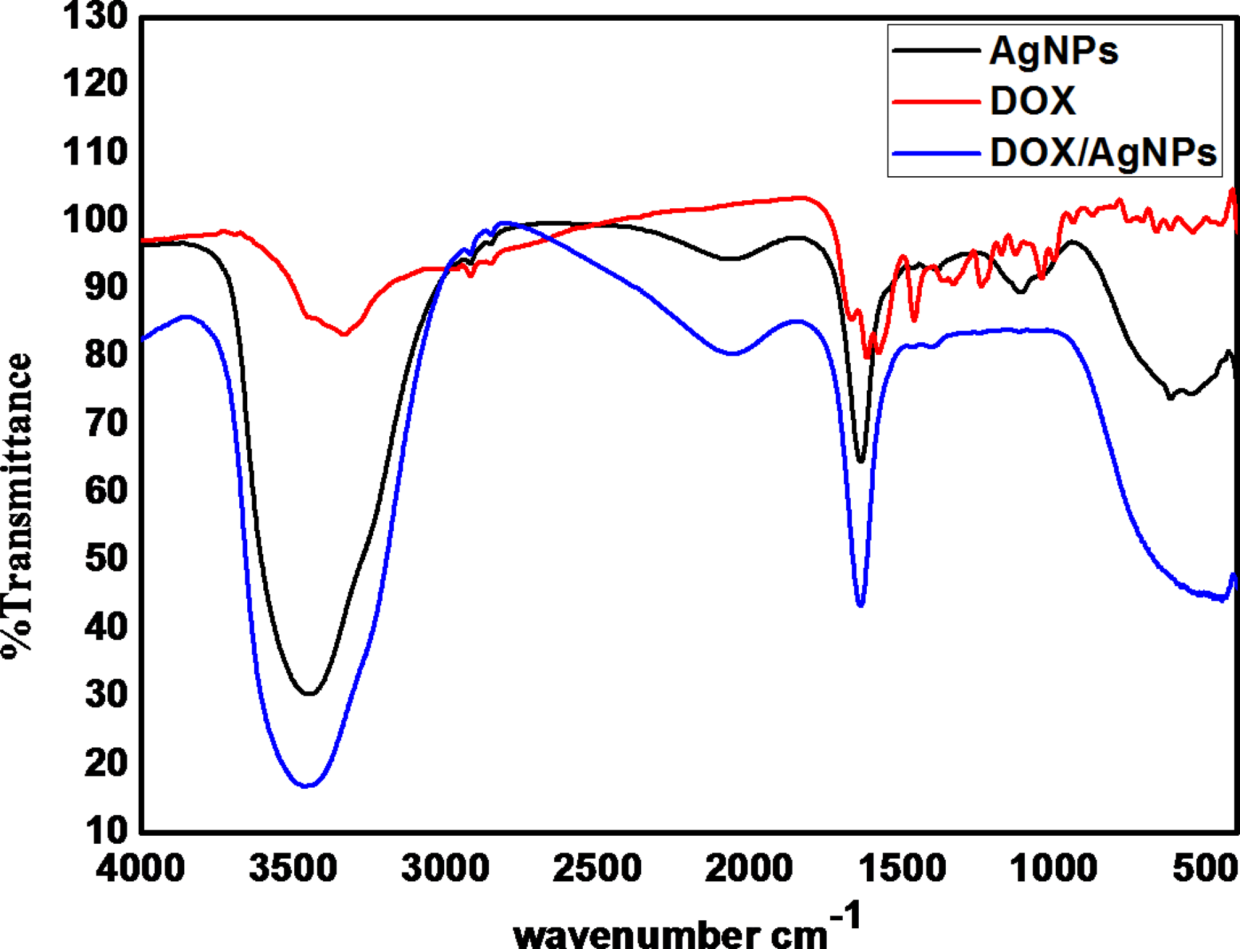
presents FTIRs of the synthesized AgNPs, Doxy and Doxy/AgNPs.

FTIRs enables the identification of organic and inorganic species in a sample by analyzing their absorption of infrared light. The interaction between chemical bonds and light involves stretching, contracting, and bending. Each chemical bond absorbs infrared light within a definite range of wavenumbers, which facilitates the determination of the functional groups present in the sample. FTIRs was used to analyze the interaction between AgNPs and Doxy as presented in Fig. 4.

In the FTIR analysis of AgNPs, the absorption peak at 3444.24 cm^−1^ related to the N–H stretching of the amino group. Absorption peak at 2920.66 cm^−1^ indicates the C–H stretching, while the peak at 1838.3 cm^−1^ signifies the C–N bond of the amide compound. Additionally, peak at 1387.53 cm^−1^ is because of the (–CH2) group, and at 1107.9 cm^−1^, it represents the C–O bond from the carboxyl group. The existence of N–H and C–N absorption is likely as a result of the interaction between the amino groups and AgNPs, where the amino groups serve as capping agents for stabilizing AgNPs^43^. The FTIR spectrum of Doxy displays a characteristic band at 3334 cm^−1^ corresponding to OH stretching vibration, along with amide bands I and II at 1616 cm^−1^ and 1578 cm^−1^, respectively. Furthermore, the vibration between 1666 cm^−1^ and 1612 cm^−1^is related to the stretching vibration of the C = O and C = C groups in the aromatic ring of doxycycline^44^. Upon loading doxycycline on AgNPs, two peak absorptions were observed in the FTIR spectra: one at 3461.6 cm^−1^, which broadened with a slight red shift, related to the carbonyl group stretch vibration of doxycycline’s aromatic ring, and the other at 1637 cm^−1^, associated with OH stretch vibration, displaying a red shift post-loading of Doxy^45^.

### Antibacterial activity of AgNPs and Doxy/AgNPs nanocomposite

Bacterial infections continue to pose a major challenge in the healthcare industry. Hence, it is imperative to create new antibiotics that possess favorable biocompatibility properties and are resistant to resistance. Silver nanoparticles show enormous potential as antimicrobial agents due to their inherent properties and remarkable thermal stability. Moreover, they exhibit minimal toxicity towards mammalian cells and tissues. One advantageous approach to developing antimicrobial nanocomposite materials is by incorporating silver with natural reductants like chitosan, dextran, sodium citrate, and ascorbic acid. The antimicrobial activity AgNPs prepared by chitosan with subsequently loading Doxy forming Doxy/AgNPs nanocomposite were determined in present work.

**Table 1:** Antimicrobial activity of AgNPs, doxycycline HCl and Doxy/AgNPs nanocomposite after 24 h of incubation time.

Sample Microorganism	Doxycycline HCl(1.9 µg/ml)	AgNPs (1.2 µg/ml)	Doxy/AgNPs nanocomposite (0.95 µg/ml)	Standard antibiotic	*P* Value
Gram negative bacteria				Gentamicin	
*Escherichia coli* *(ATCC:10536)*	37.7 ± 0.6^d^	15.3 ± 0.6^a^	21.3 ± 0.6^a^	27 ± 0.5^b^	0.000
*Klebsiella pneumonia* *(ATCC:10031)*	27.0 ± 1.0^c^	13.7 ± 0.6^a^	18.3 ± 0.6^a^	25 ± 0.5^b^	0.000
Gram positive bacteria				Ampicillin	
*Staphylococcus aureus* *(ATCC:13565)*	44.3 ± 0.6^d^	14.7 ± 0.6^a^	25.7 ± 0.6^c^	21 ± 0.1^b^	0.000
*Streptococcus mutans* *(ATCC:25175)*	36.3 ± 0.6^d^	12.3 ± 0.6^a^	22.7 ± 0.6^a^	28 ± 0.5^b^	0.000

Zone of inhibition is expressed in the form of Mean ± Standard deviation (mm), NA: No activity, well diameter (6 mm),100 µl was evaluated, Values that share the same letter at the same row are not significant. Values that share different letters at the same row are significant.

**Fig. 5:**
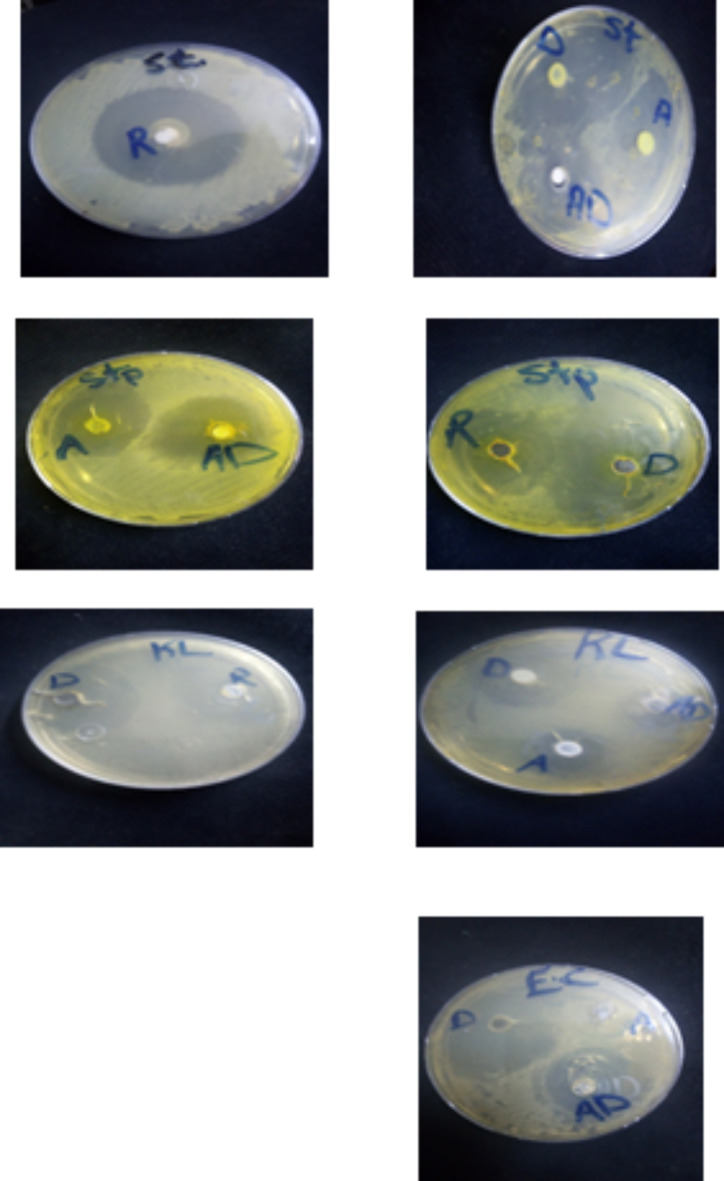
Images of disc diffusion results of AgNPs (A), Doxy (D) and Doxy loaded AgNPs(AD), (R) is stander drug on *Klebsiella pneumonia(KL)*,* Staphylococcus aureus(ST)*,* Escherichia coli(EC) and Streptococcus mutans(STP).*

In the antibacterial activity experiment, using Gram-positive *Staphylococcus aureus*, *Streptococcus mutans* and Gram-negative *Escherichia coli*, *Klebsiella pneumonia*, as models by applying agar well diffusion method after 24 h. It was observed that AgNPs prepared by chitosan and Doxy have inhibitory effect on tested bacteria. Our results is in accordance with *Eduarda Melquiades Pirette dos Santos et al. (2021)*^46^ their results demonstrated that Silver nanoparticles–chitosan composites exhibit promising antibacterial and antibiofilm therapeutic potential. Whereas the synergetic antibacterial effect of newly synthesized Doxy/AgNPs have excellent inhibitory action on *Escherichia coli*, *Klebsiella pneumonia*, *Staphylococcus aureus* and *Streptococcus mutans* than pure doxy and AgNPs, as revealed in experimental section Doxy concentration was reduced to half its original concentration in Doxy/AgNPs which mean that (0.95 µg/ml)doxy loaded on green synthesized AgNPs has an effective effect on tested bacteria as shown on Table 1; Fig. 5.In accordance with our results Ke Son Phan et al.(2022)^37^and Maheshkumar Prakash Pati et al. (2019)^47^ found that the fabricated DOX loaded AgNPs has potential antibacterial activity than the traditional form.

It has been documented that Doxy exhibits a wide range of antibacterial properties and can hinder the growing of both gram-positive and gram-negative bacteria. The mechanism behind Doxy’s antibacterial activity is believed to involve interference with protein synthesis within bacterial cells. This advantageous characteristic of Doxy is also evident in Doxy-loaded nanoparticles, which have demonstrated the ability to inhibit bacterial proliferation^48^. A potential strategy to overcome antibiotic resistance is the synergistic effects between antibiotics and NPs, which can inhibit bacteria over a long period of time. Research findings have confirmed the exceptional in vitro antibacterial efficacy of Doxy/AgNPs against both gram-positive and gram-negative bacteria. These nanoparticles accumulate on the bacterial surface and create indentations, allowing them to penetrate the cell wall and cause structural damage, leading to cell death. The bactericidal effect is further enhanced by the release of positively charged ions from the nanoparticles, which bind to the negatively charged surface of bacteria through a process called biosorption^10^. Additionally, electrostatic binding to the cell wall results in membrane depolarization, alteration of membrane potential, and loss of integrity, ultimately disrupting energy transduction and causing cell death. But, due to the peptidoglycan layer present in Gram-positive bacteria, the penetration of nanoparticles into these bacteria is more challenging, limiting their interaction to the bacterial surface only^49^.

Various mechanisms associated with anticancer and antimicrobial activities have been identified, including the direct interaction of nanoparticles (NPs) with cell membranes leading to damage, the inhibition and disruption of biofilms, the generation of both free radicals and non-radicals of reactive oxygen species (ROS) and reactive nitrogen species (RNS), the stimulation of host immune responses, and the denaturation of biological macromolecules such as nucleic acids and proteins. Among these mechanisms, the generation of ROS is particularly significant^50,51^. Nanoparticles possess significant antimicrobial potential due to their ability to generate reactive oxygen species (ROS). The generation of ROS hinders the antioxidant defense system and mechanically damages the cell membrane^52,53^. The exact mechanism by which AgNPs inhibit bacteria still indefinite, and previous researchers are under scrutiny. Three potential mechanisms for the inhibition of silver nanoparticles: (1) absorption of Ag + ions then the disruption of ATP production and DNA replication, (2) formation of ROS by Ag nanoparticles and Ag + ions, and (3) direct damage to cell membranes by Ag nanoparticles^54,55^. When nanocomposite diffuses onto bacterial media, AgNPs enter the bacterial cells and affect membrane permeability and function. Upon penetration into a cell, some particles may dissolve, releasing silver ions that, together with AgNPs form ROS and inhibit ATP production and DNA replication^56^. However, the administration of higher doses of nanoparticles (NPs) required for antibacterial efficacy may result in diminished biocompatibility and increased cytotoxicity under physiological conditions. In this context, enhancing the NP surface with additional biocompatible antibacterial agents presents a promising strategy. The incorporation of antibiotics can yield a synergistic effect when utilized alongside metal or metal oxide NPs at safe, low concentrations^57,58^. The antibacterial effect of doxycycline is enhanced when combined with AgNPs because a complex is formed in which doxycycline molecules surround an AgNPs. Consequently, the effective number of doxycycline molecules present at the site of action increases^59^.

The findings indicate that silver nanoparticles (AgNPs) disrupt the bacterial cell wall, thereby facilitating the entry of antibiotics into the bacterial cell. The combination of AgNPs with doxy increases the concentration of antibiotics, this combined approach of antibiotics and AgNPs enables the effective use of antibiotics that have become obsolete due to bacterial resistance. Such strategies present new opportunities for treating bacterial infections across healthcare, agriculture, and veterinary fields. To address the challenge posed by multidrug-resistant bacteria, there remains an urgent need for further research aimed at developing more effective and practical designs for new antimicrobial agents and pharmaceuticals for in vivo studies.

### Determination of MIC and MBC of Doxy, AgNPs and Doxy/AgNPs nanocomposite

**Table 2:** MIC and MBC of Doxy, AgNPs and Doxy/AgNPs nanocomposite.

Bacterial strains	MIC(µg/mL)			MBC(µg/mL)		
	AgNPs	Doxy	Doxy/AgNPs	AgNPs	Doxy	Doxy/AgNPs
Escherichia coli (ATCC:10536)	0.6	15.6	3.9	1.2	31.25	7.8
Klebsiella pneumonia (ATCC:10031)	1.2	31.25	15.6	2.45	62.5	15.6
Staphylococcus aureus (ATCC:13565)	0.6	7.8	3.9	1.2	15.6	7.8
Streptococcus mutans (ATCC:25175)	0.3	3.9	1.9	0.6	7.8	3.9

**Fig. 6:**
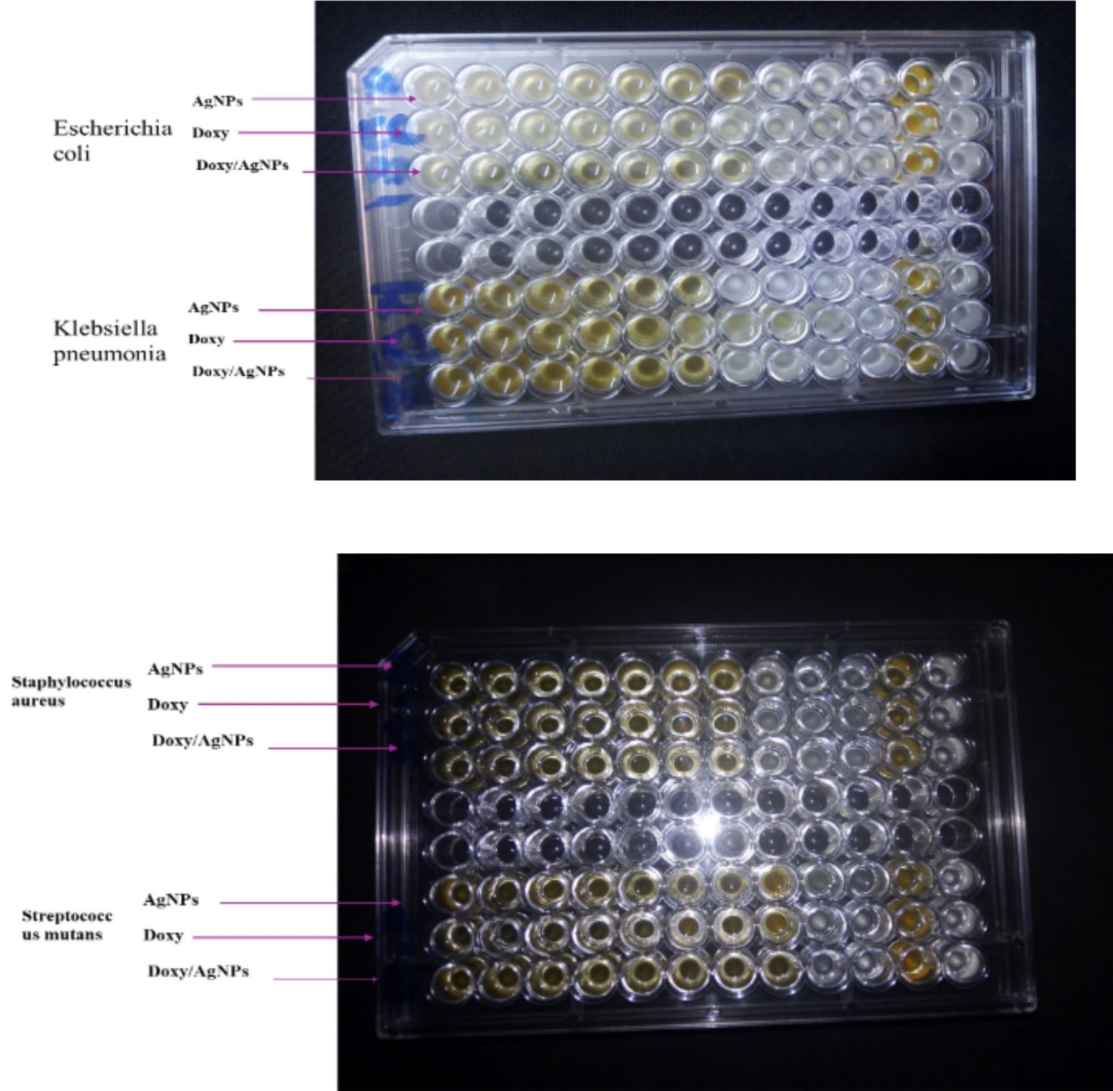
A microtiter plate for testing antibacterial activity of AgNPs, Doxy and Doxy loaded AgNPs on *Klebsiella pneumonia*,* Staphylococcus aureus*,* Escherichia coli and Streptococcus mutans.*

To determine MIC and MBC on Escherichia coli, Klebsiella pneumonia, Staphylococcus aureus and Streptococcus mutans were treated with increasing concentrations of AgNPs, Doxy and Doxy/AgNPs nanocomposite and incubated for 24 h at 37 °C. Results confirmed that AgNPs, Doxy and Doxy/AgNPs inhibit bacteria in a dose-dependent manner. Table 2; Fig. 6, shows MIC and MBC of Doxy, AgNPs and Doxy/AgNPs nanocomposite on gram positive and gram negative bacteria. Results indicate that the antibacterial effect of the Doxy/AgNPs is is more than Doxy and AgNPs alone. In addition, it is observed that the growth inhibition of Gram-positive (Staphylococcus aureus, Streptococcus mutans) bacteria inhibited at lowest concentration of Doxy, AgNPs and Doxy/AgNPs nanocomposite (MBC 15.6,7.8, 1.2,0.6,7.8 and 3.9 µg/mL respectively), while Gram-negative (Escherichia coli, Klebsiella pneumonia) bacteria inhibited at higher concentration of Doxy, AgNPs and Doxy/AgNPs nanocomposite (MBC,31.25,62.5,1.2,2.45,7.8,15.6 µg/mL respectively ) which might be due to the difference in the bacterial surface characteristics. Gram- positive and Gram-negative bacteria differ in several ways when looking at the cell wall. Gram-positive bacteria have a thick, multi-layered peptidoglycan, effectively none of lipopolysaccharide and presence of teichoic acid; Gram-negative bacteria have a thin single- layered peptidoglycan, which contains high lipopolysac- charides and absence of teichoic acid. All these different cell surface compositions make differences in the response of bacteria to NPs and antibiotics^60^. These findings are in accordance with *Maheshkumar PrakashPatil et al.*(2019)^47^. The results demonstrate that the presence of the Doxy on the AgNPs’ surface potentiates its antibacterial effects, proposing this strategy as an excellent alternative for future applications in strains resistant to this drug.

## Conclusion

Here, a simple method for colloidal AgNPs creation using chitosan as both a reducing agent and a stabilizer with subsequent loading Doxy forming Doxy/AgNPs nanocomposite exhibit promising antibacterial activity against Gram-positive and Gram-negative bacteria has been described for the first time. Results showed that doxy/AgNPs nanocomposite was successfully formed as indicated from broadening and red shift in absorption band of AgNPs from 445 nm to 450 nm after doxy addition with increase in particle size from 12 ± 2 nm to 14 ± 2 nm without effect on particle shape. The stability of the prepared doxy/AgNPs was designated via a zeta potential value of 48.5mv indicating the high stability of the prepared composite. Moreover, the enhanced antibacterial effect of doxy/AgNPs nanocomposite is twice free doxy is an indication that AgNPs can be used as drug carrier. Doxy/AgNPs nanocomposite has more inhibition zone with exceptionally low concentration 0.95 µg/ml which not effective in free doxy. MIC and MBC on *Escherichia coli*,* Klebsiella pneumonia*,* Staphylococcus aureus and Streptococcus mutans* were lower for Doxy/AgNPs nanocomposite than Doxy and AgNPs alone indicating that the presence of the Doxy on the AgNPs’ surface improve its antibacterial effects .Thus, the doxy/AgNPs developed in this study have the potential to be applied as nanotechnological therapeutic products in combating multidrug-resistant bacterial infections and bacteria growth. Future work is to use the prepared nanocomposite for laser photothermal chemotherapy combine treatment on gram-positive and gram-negative bacteria for in vivo study.

## Data Availability

“Data is provided within the manuscript”.
