# Author Correction: Reducing the effective dose of doxycycline using chitosan silver nanocomposite as a carriers on gram positive and gram-negative bacteria

**DOI:** 10.1038/s41598-025-87052-1

**Published:** 2025-02-06

**Authors:** Elham M. Mostafa, Y. Badr, M. M. Hashem, K. Abo-EL-Sooud, Amna H. Faid

**Affiliations:** 1 Department of Laser Sciences and Interactions, National Institute of Laser Enhanced Science (NILES), Cairo University, Giza, Egypt; 2 Department of Pharmacology, Faculty of Veterinary Medicine, Cairo University, Giza, Egypt

Correction to: *Scientific Reports* 10.1038/s41598-024-78326-1, published online 13 November 2024

In the original version of this Article, Reference 10 was incomplete.

Mostafa, E. M. et al. *Laser Enhanced Photothermal Effect of Silver Nanoparticles Synthesized by Chemical and Green Method on Gram-positive and Gram-negative Bacteria* (BMC Chemistry, 2024).

The full reference is listed below:

Mostafa, E. M. Laser enhanced photothermal effect of silver nanoparticles synthesized by chemical and green method on Grampositive and Gram-negative bacteria. *BMC Chem.* **18**(1), 163. 10.1186/s13065-024-01263-7 (2024).

The original Article has been corrected.

